# Single-shot full strain tensor determination with microbeam X-ray Laue diffraction and a two-dimensional energy-dispersive detector

**DOI:** 10.1107/S1600576717005581

**Published:** 2017-05-30

**Authors:** A. Abboud, C. Kirchlechner, J. Keckes, T. Conka Nurdan, S. Send, J. S. Micha, O. Ulrich, R. Hartmann, L. Strüder, U. Pietsch

**Affiliations:** a Department of Physics, University of Siegen, Siegen 57072, Germany; b Max-Planck-Institut für Eisenforschung GmbH, Düsseldorf 40237, Germany; c Montanuniversität Leoben, Leoben 8700, Austria; d Fakultät für Ingenieurwissenschaften, Türkish German Universität, Sahinkaya Caddesi 86, Istanbul, 34820, Turkey; e CEA-Grenoble/DRFMC/SprAM, 17 rue des Martyrs, Grenoble Cedex 9, F-38054, France; f PNSensor GmbH, Otto-Hahn-Ring 6, München 81739, Germany

**Keywords:** strain, microbeam X-ray Laue diffraction, energy-dispersive X-ray detectors

## Abstract

The full strain and stress tensor determination in a triaxially stressed single crystal using X-ray diffraction requires a series of lattice spacing measurements at different crystal orientations. This can be achieved using a tunable X-ray source. This article reports on a novel experimental procedure for single-shot full strain tensor determination using polychromatic synchrotron radiation with an energy range from 5 to 23 keV. Microbeam X-ray Laue diffraction patterns were collected from a copper micro-bending beam along the central axis (centroid of the cross section). Taking advantage of a two-dimensional energy-dispersive X-ray detector (pnCCD), the position and energy of the collected Laue spots were measured for multiple positions on the sample, allowing the measurement of variations in the local microstructure. At the same time, both the deviatoric and hydrostatic components of the elastic strain and stress tensors were calculated.

## Abstract 2

By simultaneously measuring changes in energy and reflection angle of Laue spots with respect to a reference position, it is possible to measure all lattice parameters of a unit cell and calculate the full strain/stress tensors in a single-shot experiment with high spatial resolution.

## 1. Introduction   

The mechanical properties of micrometre-sized materials, scaling from 0.1 to 10 µm, are of increasing importance owing to current developments in micro systems technology, nano-electromechanical systems and downsized medical devices such as ‘implantable medical devices’. In the past few decades, it has been discovered that the mechanical properties might change if the size of the specimen is reduced to the micrometre or nanometre scale (Fleck *et al.*, 1994 ▸; Stölken & Evans, 1998 ▸; Haque & Saif, 2003 ▸; Uchic *et al.*, 2004 ▸), driving further investigations. This includes structure analysis of materials on the micrometre scale.

Strain analysis at the micrometre level by X-ray diffraction techniques requires a fixed beam footprint on the sample during the whole measurement time. Any rotational movement of the sample would change the volume under investigation; therefore, ideally a single-shot experiment is performed.

Although the basics for determining the full strain tensor components, both the deviatoric and hydrostatic parts, conceptually are well understood (Chung & Ice, 1999 ▸; Busing & Levy, 1967 ▸; Rollett, 1965 ▸), there has been no experimental realization of a single-shot measurement.

Advances in synchrotron X-ray techniques (Tamura *et al.*, 2000 ▸; Liu *et al.*, 2004 ▸) have shown that strain measurements under a fixed angle of incidence with respect to the sample are possible. However, one has to rotate the detector angle and switch between mono- and polychromatic X-rays in order to resolve the Laue patterns and determine the full strain tensor. On the other hand, experiments at BM32 of the European Synchrotron Radiation Facility (ESRF) (Robach *et al.*, 2012 ▸) used a single-crystal diamond filter to measure the energy profiles of Laue spots with improved resolution. More recently, full elastic stress and strain tensor measurements were performed on a copper through-silicon via with a combination of mono- and polychromatic X-rays to scan the energy of Laue spots (Levine *et al.*, 2015 ▸). These studies provide ground breaking results. However, switching and tuning the X-ray beam is a time-consuming procedure that does not fully guarantee a fixed footprint of the beam on the sample, which prohibits the application of this approach for *in situ* sub-micrometre full strain measurements.

Energy-dispersive Laue diffraction (EDLD) using polychromatic synchrotron radiation (Send *et al.*, 2012 ▸; Abboud *et al.*, 2014 ▸) and a two-dimensional energy-dispersive detector (Strüder *et al.*, 2001 ▸) combined with state of the art focusing optics (Ulrich *et al.*, 2011 ▸) offer outstanding advantages over the aforementioned techniques. As demonstrated recently (Abboud *et al.*, 2014 ▸), EDLD can be successfully applied to quantify defects in a single-crystal copper micropillar. Using a pnCCD detector and a sub-micrometre-focused X-ray beam, the single-shot-recorded Laue patterns contain both the intensity distribution and the position and energy information necessary to calculate the full strain tensor without the need of any rotation. By following the energy distribution along a streaked Laue spot, the type of lattice deformation in the sample was determined. This novel approach was limited by measuring the position and energy of only two Laue reflections (linearly dependent), and thus the full strain tensor could not be calculated.

By measuring at least three independent reflections (or the energy of two independent reflections), EDLD can be extended to measure the full strain tensor (Busing & Levy, 1967 ▸; Chung & Ice, 1999 ▸). In this article we report the measurement of the full strain tensor by EDLD. Here the experimental technique was tested on a deformed copper micro-bending beam where the three-dimensional structure, *i.e.* the crystal orientation and the six unit-cell parameters, was determined. By measuring changes in energy and reflection angle of Laue spots with respect to a reference point we could measure all lattice parameters of the unit cell and calculate the full strain (stress) tensor components at multiple points along the specimen central axis.

## 2. Experimental procedure   

**Figure 1:**
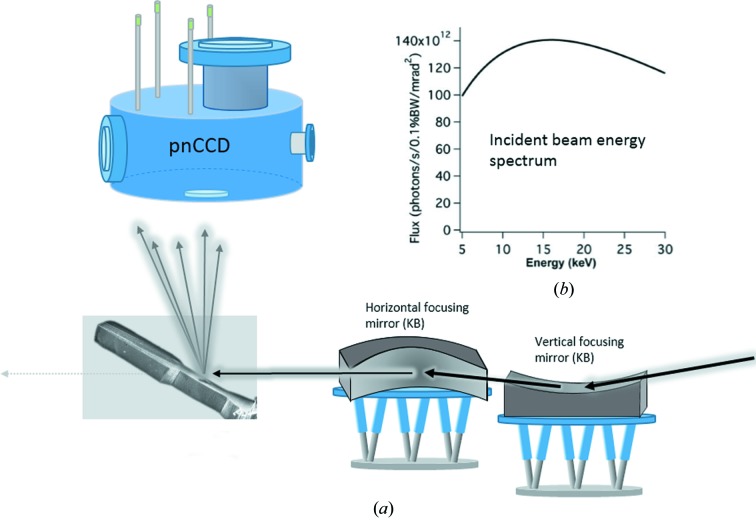
(*a*) A sketch of the geometry of the experimental setup at the BM32 beamline of the ESRF. Two KB mirrors were used to focus the incident beam onto the sample. The pnCCD detector was mounted facing down on the sample. (*b*) The energy spectrum of the X-rays used.

The EDLD experiment was performed using the microbeam Laue diffraction setup of the CRG-IF BM32 beamline at ESRF (Ulrich *et al.*, 2011 ▸). The primary beam provides polychromatic X-ray photons ranging from 5 to 23 keV. With the help of two Kirkpatrick–Baez (KB) mirrors (Yumoto *et al.*, 2013 ▸) (Fig. 1 ▸), the beam size was reduced to 0.5 and 0.8 µm (FWHM) in the vertical and horizontal directions, respectively.

**Figure 2:**
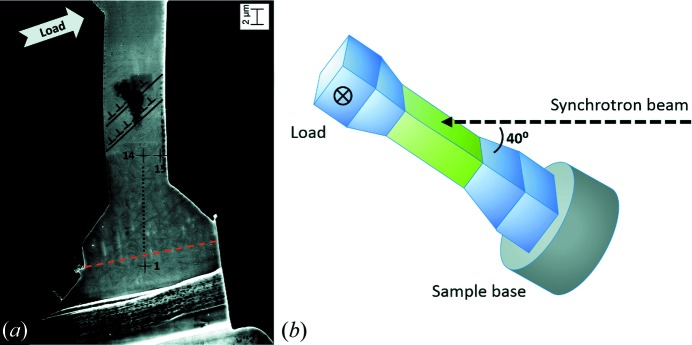
(*a*) Scanning electron microscopy image of the micro-bending beam of copper used in the experiment. The sample has been loaded in the [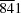] direction. Dots along the dotted line indicate the measurement points. (*b*) Sketch of the sample with respect to the incident X-ray beam.

The sample used for the present experiment is a copper single crystal shaped by means of a focused ion beam (Zeiss 1540XB) following the approach of Moser *et al.* (2012 ▸) and Kapp *et al.* (2015 ▸). The specimen is shown in Fig. 2 ▸. It has a gauge length of 20 µm in height and widths of 7 and 9 µm at the center. Prior to this experiment, the sample was milled with the [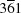] crystallographic axis parallel to the central axis and the [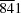] crystallographic direction parallel to the loading axis. A Hysitron PicoIndenter PI 85 (Hysitron, Minneapolis, MN, USA) with a nominal force resolution of 0.1 µN equipped with a doped diamond Berkovich indenter tip was used for the bending experiment.

An energy-dispersive pnCCD detector was used to acquire the data. Its active volume is made from a 450 µm weakly doped n-type silicon. The front side is divided into 384 × 384 pixels, each of 75 × 75 µm in size. The spatial resolution is limited by the pixel size but can be further reduced by dedicated algorithms (Abboud *et al.*, 2013 ▸; Soltau *et al.*, 2014 ▸), while the energy resolution (FWHM) is limited by the electronic noise and the Fano limit of silicon (Fano, 1947 ▸). The latter is measured to be 136.5 eV at 8040 eV. Further details on this type of pnCCD are given by Send *et al.* (2013 ▸) and Abboud *et al.* (2014 ▸).

Laue diffraction patterns were collected in reflection geometry using the pnCCD installed in a position nearly perpendicular to the incident beam (Fig. 1 ▸). The micro-bending beam was scanned relative to the focused X-ray beam from the base upward, as shown in Fig. 2 ▸(*a*), with a step size of 1 µm, starting from below the dashed line (reference measurement) and following the central axis of the beam. The last measurement was performed at the edge, 2 µm from position 14 (Fig. 2 ▸ *a*). In total we probed 15 positions using the single-photon counting mode of the detector, *i.e.* at each position 50 000 frames were recorded with a frame rate of 92 frames per second. Fig. 2 ▸(*b*) shows a two-dimensional sketch of the sample and its position relative to the incident X-ray beam.

## 3. Analysis method   

**Figure 3:**
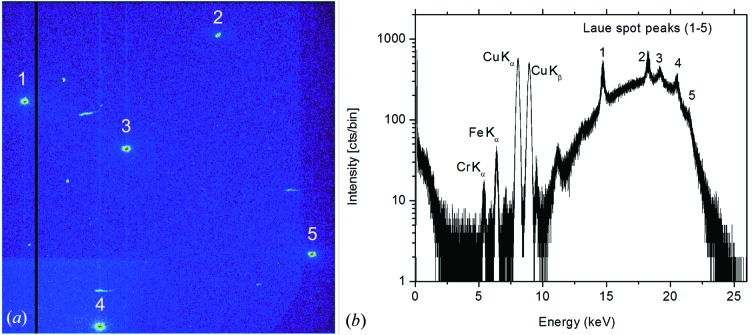
(*a*) Two-dimensional intensity map with five visible Laue spots from the measurement at position 1 (reference) on the sample. (*b*) Corresponding energy spectrum showing the Laue spots’ energy distributions and the fluorescence radiation from the sample (Cu) and detector housing (Fe and Cr), which are taken as background radiation.

After post analysis (noise, offset, event recombination *etc.*; Andritschke *et al.*, 2008 ▸) of the recorded data, three relevant parameters were extracted for further analysis. These are the position coordinates (*x*, *y*) and the energy of every recombined (photon) event. Fig. 3 ▸(*a*) shows the energy-integrated intensity image containing five Laue spots. In Fig. 3 ▸(*b*) an energy histogram of all events is shown. The copper fluorescence originates from the sample, and the iron and chromium fluorescence from the detector housing. Peaks numbered from 1 to 5 represent the Laue spots’ energy peaks overlaying the background hump of the primary X-ray beam.

The open-source *LaueTools* (Micha, 2010 ▸) software was used to associate the Laue spots with the corresponding Miller indices. The spatial intensity distribution of each Laue spot was fitted with a two-dimensional Gaussian to find the spot center (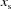, 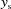). Gaussian fitting was also performed on the energy histogram of each Laue spot (10 × 10 pixels) to determine the spot energy. Bragg angles were then calculated and all the values are summarized in Table 1 ▸.

### 3.1. Calculation of unit-cell parameters   

**Figure 4:**
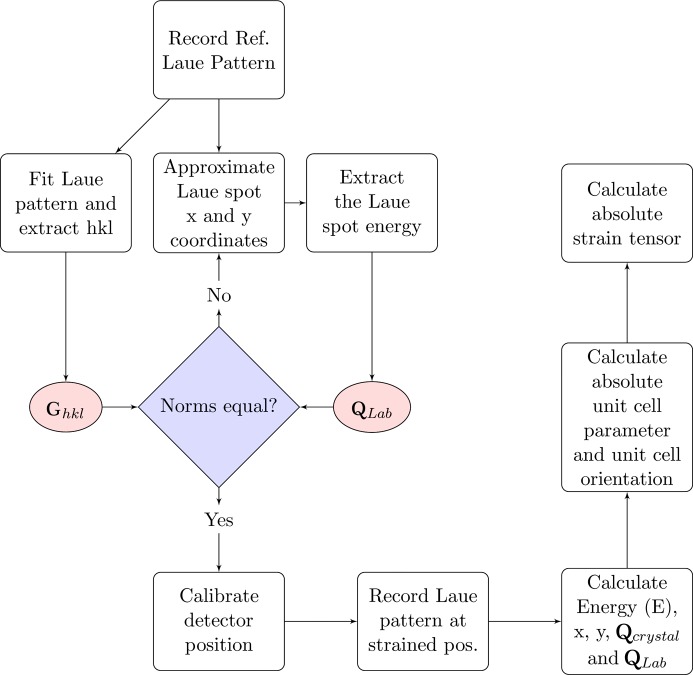
Flow chart for calibrating the position of the detector and calculating the strain tensor using an energy-dispersive two-dimensional detector. The chart is an extension of the work of Chung & Ice (1999 ▸). Details are provided in the text.

**Figure 5:**
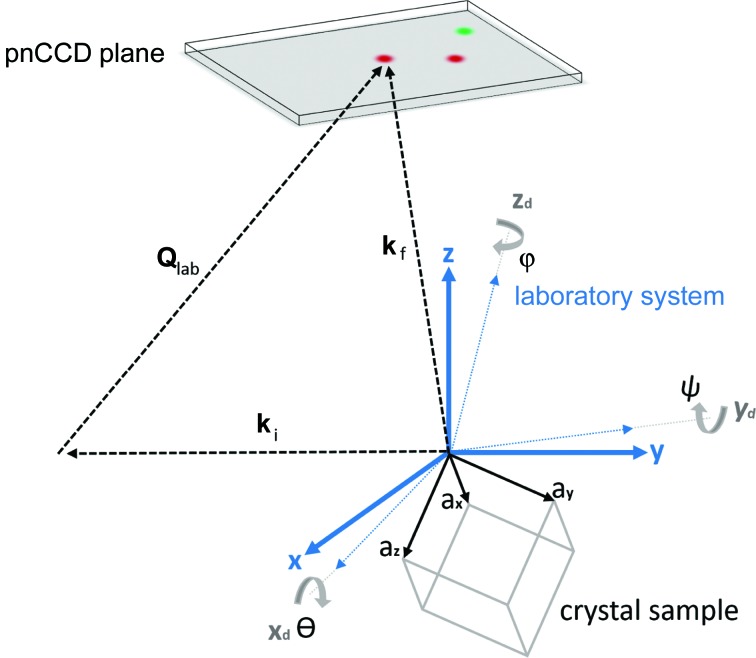
The experiment geometry. For every Laue spot on the pnCCD, a scattering vector 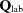 is assigned. This vector is described either in the crystal frame through the Miller indices as 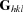 or in the laboratory-frame coordinate system by the measured position and energy of the Laue spot. Angles (φ, θ, ψ) were used to correct the tilt of the detector frame (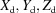) with respect to the laboratory frame (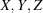).

The process of calculating the components of the full strain tensor of a sample using EDLD and a pnCCD is schematically outlined in the chart of Fig. 4 ▸. Using the setup sketched in Fig. 5 ▸, the broad-bandpass microbeam illuminates the copper sample over an area determined by the beam size, producing Laue spots. From our knowledge of the Laue spots’ positions and energies (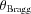 and *E*), a scattering vector 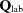 is defined for every Laue spot as the difference between the scattering wavevector 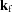 and the incident wavevector 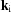, as shown in equation (1) and in Fig. 5 ▸:
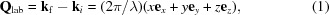
where 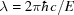 with 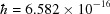
 eV (Planck constant) and 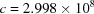
 m s^−1^ (speed of light).

At the same time, a reciprocal lattice vector 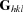 is defined for every set of real lattice planes, with Miller indices (*hkl*), using the reciprocal lattice vectors 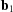, 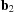 and 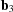: 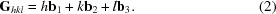
The magnitude of the scattering vector 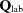 is equal to the magnitude of the reciprocal lattice vector 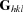 through the relations shown in equation (3):
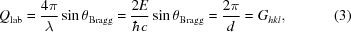
where *d* is the inter-planer spacing distance referred to a lattice plane (*hkl*). Moreover, the scattering vector is oriented perpendicular to the reflecting lattice plane, whereby 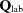 becomes equal to the reciprocal lattice vector of the crystal:
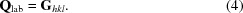
Using equation (4), we calculate the reciprocal unit-cell vectors (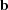) by solving equation (5): 
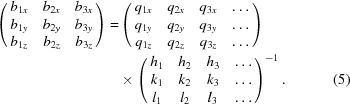
Once the reciprocal vectors 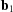, 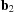 and 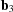 have been obtained from the pnCCD data sets, the corresponding basis vectors can be calculated using the reverse transformation shown in equation (6): 
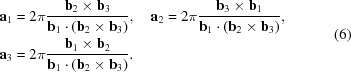



### 3.2. Calculation of full strain and stress tensors   

The full strain tensor of a crystal is composed of a deviatoric and a hydrostatic part as shown in equation (7), where 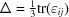 is the isostatic strain defined as the mean strain component along the diagonal axis of the full strain tensor (deviatoric part): 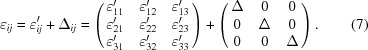
The deviatoric term is related to the deformation of the unit cell at constant volume, while the hydrostatic term corresponds to a change in volume, without angle variation.

Using an approach similar to that adopted by Chung & Ice (1999 ▸), the infinitesimal (Lagrangian) strain components were directly obtained using
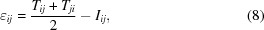
where 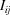 is the unit matrix and *T* is the transformation matrix which maps the unstrained (reference) to the strained vectors as in equation (9):
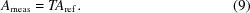
In this form, *A* is defined, in equation (10), as the transformation matrix of any vector in the crystal from unit-cell to Cartesian coordinates given by 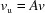:
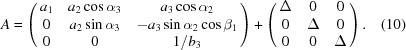
Here 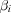 and 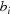 are reciprocal lattice parameters and 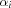 and 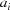 are real lattice parameters of the unit cell in direct space.

### 3.3. Uncertainties   

Uncertainties in calculating the lattice parameters and strain and stress components originate from two main sources. The first is the uncertainties in the Bragg angle and the energy of Laue spots. The second is the residual strain in the reference point of measurement, *i.e.* the calibration point.

The scattering angles of the Laue spots were calculated by measuring the sample-to-detector distance (STD) and the coordinates of the spots in the detector plane. Respective pixel positions were extracted from a two-dimensional Gaussian fit of the Laue spot’s intensity profile. One standard deviation was considered as the uncertainty value of the Laue spots’ centers, which is on average less than half a pixel, as well as for the uncertainty calculations of the Laue spots’ energies estimated by equation (12), where the FWHM(*E*) is obtained from the Gaussian fit of the energy histogram. For all measured Laue spots, the standard deviation was on average less than 150 eV.

To reduce the geometric uncertainties (distances) we follow the logic presented in Fig. 4 ▸. A reference Laue pattern was recorded and fitted using *LaueTools* to determine the Miller indices. By comparing the magnitude and orientation (angle) of the measured vector (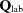) with those of the fit (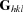), for every Laue spot, the uncertainty in the STD is reduced to 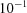. Once the Bragg angle and energy of the Laue spots are fixed, we solve equation (5) and calculate an initial set of lattice parameters for the calibration point.

**Figure 6:**
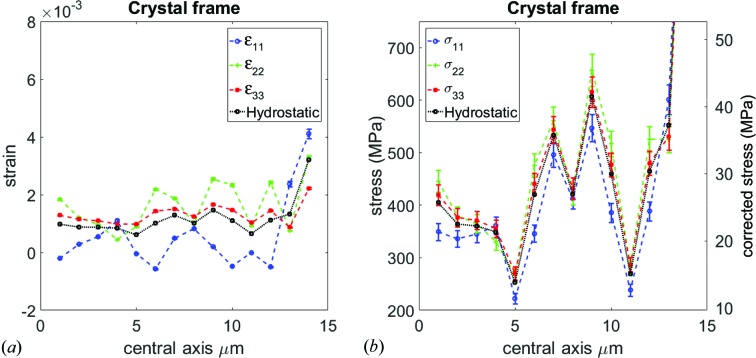
(*a*) Normal strain components 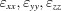, plotted as a function of distance along the central axis. The uncertainties are one standard deviation. The sawtooth pattern can be interpreted as the presence of a strain gradient along the central axis (see Fig. 8 ▸). (*b*) The strain pattern translated to the normal stress components, showing a similar response. The right-hand *y* axis shows the corrected stress values after taking into account the uncertainty in the calibration measurement. The last stress value is around 100 MPa.

Step two required correcting for the relative tilts (φ, θ, ψ) between the detector plane and the laboratory-frame coordinate system as shown in Fig. 5 ▸. In this step we initiated a set of random tilt angles and incremented the tilt in steps of 0.001 rad, where in each step we reduce the difference between the calculated and literature values of the lattice parameters of copper. The tilt angles were calculated to be −0.01, −0.01 and −0.04 rad. The transformation matrix *M* of equation (11) was used to correct all vectors measured in the laboratory-frame coordinate system: 
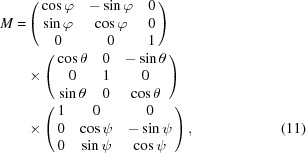


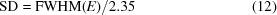
(SD being the standard deviation). The uncertainties discussed above must be propagated through the analysis in order to estimate uncertainties for the unit-cell parameters and the components of the strain and stress tensors. The uncertainties of the evaluated stress tensor components contain uncertainties of the elastic constants for copper at room temperature, given by 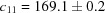
 GPa, 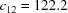 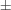 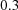
 GPa and 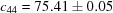
 GPa (Ledbetter & Naimon, 1974 ▸). The calculated error bars are shown alongside the strain and stress values in Figs. 6 ▸ and 7 ▸.

Strain in the sample at the position of the calibration point is the second source of uncertainty and would certainly add a systematic offset to the evaluated strain values in all of the following measurements. In order to determine this offset, we simulated the consequence of an artificial strain added to the calibration point by multiplying the lattice vectors with a constant factor, thereby resulting in a slight increase in volume of the unit cell (volumetric strain). As an example, a strain of 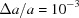 (present at the calibration point) would increase the stress amplitude of any subsequent measurement points by approximately one order of magnitude. As it turned out during data analysis, the calibration point that was used in the calculation was not strain free. Hence the relative stress values must be corrected to obtain their correct magnitudes.

### 3.4. Euler angle transformation   

The crystallographic orientation of the measured sample is calculated in the laboratory-frame coordinate system and is transformed into the crystal-frame coordinate system by a three-axis Euler angular rotation following the (φ_Euler_, θ_Euler_, ψ_Euler_) convention described by Goldstein (1980 ▸). The unit cell is first aligned with the [100], [010] and [001] directions along the *x*, *y* and *z* axes of the laboratory-frame coordinate system, respectively. With steps of 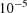 radians, the unit cell is rotated counterclockwise by φ about the *z* axis (taking *xyz* to *x*1 *y*1 *z*), then rotated counterclockwise by θ about *x*1 (*x*1 *y*1 *z* to *x*1 *y*2 *z*2) and, finally, counterclockwise by ψ about *z*2 (*x*1 *y*2 *z*2 to *x*3 *y*3 *z*2). All rotations are described by the matrix *E* given in equation (13):
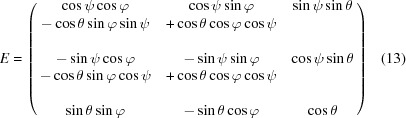
where the inverse is 
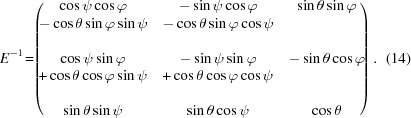
The resulting Euler angles are (−1.2545, 0.9551, 2.0479) 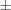 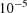
 rad, respectively. The rotation matrix 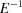 is used to transfer vectors from the laboratory frame into the crystal frame of reference.

## 4. Results and discussion   

The method introduced by Chung & Ice (1999 ▸) and Busing & Levy (1967 ▸) is applied to EDLD and is illustrated in an example on a deformed copper sample. The reciprocal lattice vectors (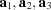) are calculated at the reference point [position 1 in Fig. 2 ▸(*a*)] by solving the overdetermined matrix of equation (5) and transforming the obtained basis into real-space coordinate. The result is reported in equation (15):
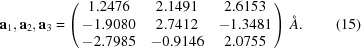



The magnitudes of the lattice parameters (Å) and the corresponding cell angles (°) are shown in equation (16):
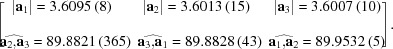


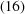
The values in equation (16) verify the cubic symmetry and show that the lattice parameters of copper can be obtained with an accuracy of 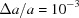 (Davey, 1925 ▸; Wyckoff, 1963 ▸). This deviation in the lattice parameters at the calibration point needs to be taken into account as discussed in §3.3. The unit-cell parameters are calculated at different positions along the microbeam axis. These values are then used in the calculation described in §3.2, resulting in the full strain and stress tensors. As an example, the strain at position number 2 is given in equation (17), presented in the crystal-frame coordinate system: 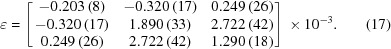
The isostatic term is calculated to be 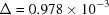.

**Figure 8:**
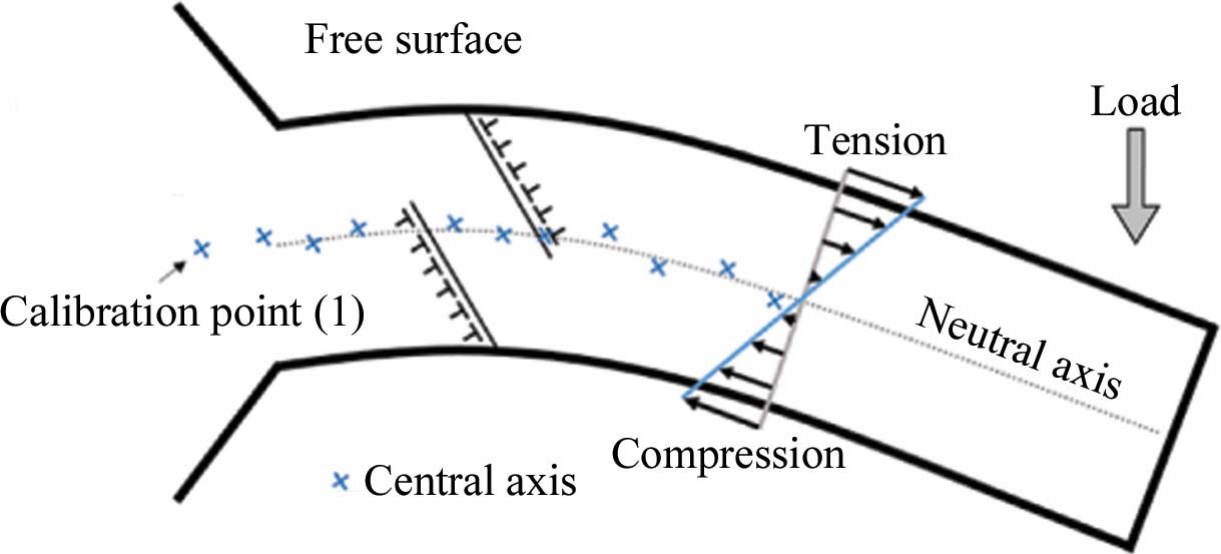
A simplified sketch of the bending beam and two glide planes (one with positive and one with negative Burger’s vectors). The dotted line shows the expected location of the neutral axis, and the crosses show the measurement points, *i.e.* possible deviation from the neutral axis.

Figs. 6 ▸ and 7 ▸ show all the calculated strain and stress tensor components taken along the central axis of the copper cantilever along with the calculated uncertainties. Before interpreting the data, we need to highlight points that affect, qualitatively and quantitatively, the strain and stress values:

(1) The collected Laue spots have a spectral distribution between 14.6 and 21.3 keV. This means that different reflections probe different depths inside the sample. Moreover, the incident X-ray beam was oriented at an angle of 40° with respect to the sample. Therefore, the penetration depth is around 5 µm beneath the surface and the *sampled* volume, given by the path of the X-ray beam through the sample, is approximately 16 µm. As a result, the strain and stress tensor values would be an average of the sampled volume.

(2) The X-ray beam was centered in the middle of the micro-bending beam by performing a cross-sectional scan of the sample and detecting its edges by the excited fluorescence photons by means of an energy-dispersive point detector. This process was repeated every time the sample was translated along its microbeam parallel to the neutral axis. This means that drifts of the X-ray beam from the neutral axis are expected and the scan follows the central axis instead (Fig. 8 ▸).

(3) The presence of strain in the calibration point would lead to an offset of the absolute stress values.

(4) In the micro-bending beam, dislocations are piled up at the neutral axis. In such pileup, the shear stress on the leading dislocation of the pileup is the globally applied shear stress (at maximum, half of the global normal stress) multiplied by the number of dislocations. This can exceed the global (and local) normal stresses.

(5) For micro-sized samples, the sample geometry (length and aspect ratio) causes an increase in the yield stress of the material. Although the nature of this effect is still under discussion, there have been many reports in the direction of pronounced hardening and strong size effects in single crystals (Kiener *et al.*, 2008 ▸).

Figs. 6 ▸ and 7 ▸ show the change of the strain and stress along the central axis. The stress figures are presented with a double *y* axis. The left one is directly calculated from the strain, while in the right axis the residual strain in the calibration point was taken into account as discussed in §3.3.

Taking into account points 1–5 mentioned above, the strain amplitudes in Figs. 6 ▸(*a*) and 7 ▸(*a*) can be explained to be changing as the X-ray beam position with respect to the neutral axis changes. This scenario is sketched in Fig. 8 ▸. The neutral axis is defined as the line where the strain (and consequently stress) is equal to zero. However, slip systems, which are part of the underlying dislocation mechanism for the deformation (Kapp *et al.*, 2015 ▸) of these microbeams, lead to formation of dislocations at either side of the bending beam. Most of these dislocations will be trapped and only a minority will penetrate to the other side, because the neutral axis of the beam acts as a strain interface for dislocation motion. This trapping effect produces large strain gradients of opposite signs which are visible as fluctuations (up and down trends) of the strain and consequently the stress values in Figs. 6 ▸(*b*) and 7 ▸(*b*).

On the other hand, closer to the free surface of the sample, *i.e.* away from the central axis, dislocations are trapped, and increases in the values of the strain and stress magnitudes are seen at the last scan positions in Figs. 6 ▸ and 7 ▸, where the stress shoots to above 100 MPa. All the stress values obtained are below the tensile strength of pure copper, measured to be between 224 and 314 MPa (Goodfellow Catalogue, 1993–1994 ▸).

## 5. Summary and conclusions   

We have described and demonstrated a new measurement technique by applying polychromatic microbeam X-ray diffraction to determine the crystallographic orientation of a strained single-crystalline copper micro-bending beam. Using a two-dimensional energy-dispersive detector, the EDLD method allows for simultaneous measurement of diffraction peak angles and energies without any rotation of the sample with respect to the incident beam.

In this particular example, the calibration point was not strain free. In the optimal case, this can be bypassed by selecting an unstrained position on the sample surface which leads to an accurate data interpretation.

Relative to a reference measurement point on the sample, one is able to determine all components of the full strain tensor in a single-shot experiment. The strain and stress tensor component variations along the bending beam show strain gradients due to heterogeneous distributions of dislocations along the central axis.

Presently, the experimental uncertainty is limited by the relatively small number of collected Laue spots and the precision in determining their position and energy. While the first can be improved by collecting a larger number of spots (in this case a larger detector module would solve the problem), the later depends on the energy resolution and position resolution of the detector. The energy resolution of the pnCCD is already at the theoretical limit of what is possible with silicon (Fano limit). On the other hand, the precision in determining the centroid of the Laue spots increases by improving the peak fitting routines and implementing sub-pixel event position reconstruction. Another improvement of the spatial resolution would be to use digital image correlation techniques (Petit *et al.*, 2015 ▸), which bypass the need for the exact position of the Laue spots’ centers.

One can also improve the resolution by reducing the sampling volume of the probed specimen by reducing the sample thickness.

Measuring the full strain tensor in a single-shot non-destructive experiment provides numerous advantages for the analysis of technical materials and devices. Stresses and strains are the primary causes of structural failure. The possibility to realize a fast two-dimensional screening procedure of imperfection in single- and polycrystalline samples by means of a nanobeam X-ray source allows us to determine, for example, the early stages of crack formation, whisker growth and delamination in composite materials.

The method introduced is robust and appropriate for *in situ* applications and can be extended to polycrystalline materials with high spatial resolution.

## Figures and Tables

**Figure 7:**
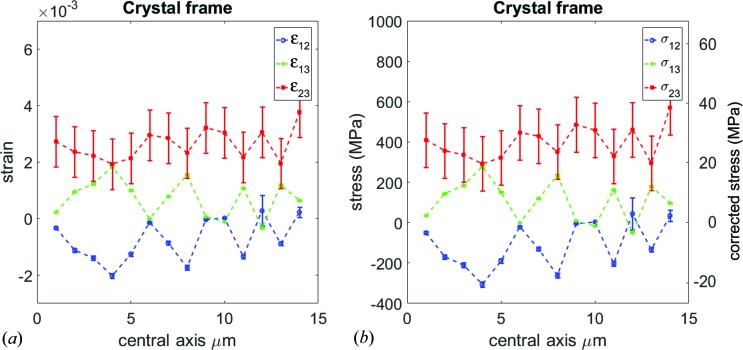
(*a*) Shear strain and (*b*) stress tensor components are plotted as a function of distance along the central axis.

**Table 1:** Measured Laue spot energies and FWHMs, the calculated Bragg angle (θ), and the evaluated Miller indices for the reference position

Spot No.	Energy (eV)	FWHM (eV)	2θ (°)	*hkl*
1	14608.44	282.68	92.530 (5)	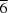 20
2	18143.30	296.79	95.125 (5)	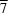 31
3	19079.14	353.12	88.230 (5)	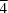 20
4	20389.52	364.50	96.284 (5)	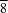 20
5	21360.79	425.78	99.844 (5)	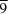 3 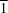
